# Detection of Nonsynonymous Single Variants in Human HLA-DRB1 Exon 2 Associated with Renal Transplant Rejection

**DOI:** 10.3390/medicina59061116

**Published:** 2023-06-09

**Authors:** Mohamed M. Hassan, Mohamed A. Hussain, Sababil S. Ali, Mohammed A. Mahdi, Nouh Saad Mohamed, Hanadi AbdElbagi, Osama Mohamed, Asmaa E. Sherif, Wadah Osman, Sabrin R. M. Ibrahim, Kholoud F. Ghazawi, Samar F. Miski, Gamal A. Mohamed, Ahmed Ashour

**Affiliations:** 1 Department of Hematology, Faculty of Medical Laboratory Sciences, National University, Khartoum 11111, Sudan; 2 Department of Pharmaceutical Microbiology, Faculty of Pharmacy, International University of Africa, Khartoum 11111, Sudan; mkasamber@gmail.com; 3 Department of Parasitology and Medical Entomology, Faculty of Medical Laboratory Sciences, National University, Khartoum11111, Sudan; sababil990@gmail.com; 4 Department of Chemical Pathology, Faculty of Medical Laboratory Sciences, National University, Khartoum 11111, Sudan; mohamed.gadir@yahoo.com; 5 Molecular Biology Unit, Sirius Training and Research Centre, Khartoum 11111, Sudan; nouh_saad@outlook.com (N.S.M.); hanadi3814@gmail.com (H.A.); 6 Department of Molecular Biology, National University Biomedical Research Institute, National University, Khartoum 11111, Sudan; osama.mohammed4@gmail.com; 7 Department of Pharmacognosy, Faculty of Pharmacy, Prince Sattam Bin Abdulaziz University, Al-kharj 11942, Saudi Arabia; ae.sherif@psau.edu.sa (A.E.S.); w.osman@psau.edu.sa (W.O.); ahmedadelashour@yahoo.com (A.A.); 8 Department of Pharmacognosy, Faculty of Pharmacy, Mansoura University, Mansoura 35516, Egypt; 9 Department of Pharmacognosy, Faculty of Pharmacy, University of Khartoum, Al-Qasr Ave, Khartoum 11111, Sudan; 10 Preparatory Year Program, Department of Chemistry, Batterjee Medical College, Jeddah 21442, Saudi Arabia; sabrin.ibrahim@bmc.edu.sa; 11 Department of Pharmacognosy, Faculty of Pharmacy, Assiut University, Assiut 71526, Egypt; 12 Clinical Pharmacy Department, College of Pharmacy, Umm Al-Qura University, Makkah 24382, Saudi Arabia; kfghazawi@uqu.edu.sa; 13 Department of Pharmacology and Toxicology, College of Pharmacy, Taibah University, Al-Madinah Al-Munawwarah 30078, Saudi Arabia; smiski@taibahu.edu.sa; 14 Department of Natural Products and Alternative Medicine, Faculty of Pharmacy, King Abdulaziz University, Jeddah 21589, Saudi Arabia; gahussein@kau.edu.sa

**Keywords:** graft rejection, HLA-DRB1 gene, renal diseases, single nucleotide variants snvs, DNA sequencing, HLA typing, health and wellbeing

## Abstract

*Background*: HLA-DRB1 is the most polymorphic gene in the human leukocyte antigen (HLA) class II, and exon 2 is critical because it encodes antigen-binding sites. This study aimed to detect functional or marker genetic variants of HLA-DRB1 exon 2 in renal transplant recipients (acceptance and rejection) using Sanger sequencing. *Methods*: This hospital-based case-control study collected samples from two hospitals over seven months. The 60 participants were equally divided into three groups: rejection, acceptance, and control. The target regions were amplified and sequenced by PCR and Sanger sequencing. Several bioinformatics tools have been used to assess the impact of non-synonymous single-nucleotide variants (nsSNVs) on protein function and structure. The sequences data that support the findings of this study with accession numbers (OQ747803-OQ747862) are available in National Center for Biotechnology Information (GenBank database). *Results*: Seven SNVs were identified, two of which were novel (chr6(GRCh38.p12): 32584356C>A (K41N) and 32584113C>A (R122R)). Three of the seven SNVs were non-synonymous and found in the rejection group (chr6(GRCh38.p12): 32584356C>A (K41N), 32584304A>G (Y59H), and 32584152T>A (R109S)). The nsSNVs had varying effects on protein function, structure, and physicochemical parameters and could play a role in renal transplant rejection. The chr6(GRCh38.p12):32584152T>A variant showed the greatest impact. This is because of its conserved nature, main domain location, and pathogenic effects on protein structure, function, and stability. Finally, no significant markers were identified in the acceptance samples. *Conclusion:* Pathogenic variants can affect intramolecular/intermolecular interactions of amino acid residues, protein function/structure, and disease risk. HLA typing based on functional SNVs could be a comprehensive, accurate, and low-cost method for covering all HLA genes while shedding light on previously unknown causes in many graft rejection cases.

## 1. Introduction

Over 800 million people worldwide (10% of the population) have chronic renal disease (CRD) [1]. CRD is more common in older people, women, and people with diabetes and high blood pressure [2]. Low- and middle-income countries face a significant burden of CRD [3,4]. Chronic renal disease is one of the leading causes of death worldwide [1]. During the period 1990–2017, CRD mortality increased by 41.5% globally [5]. Over one million patients were predicted to have end-stage renal disease (ESRD) globally two decades ago, with a 7% annual increase [6]. In Sudan, the prevalence of CRD ranges from 7.7 to 11%, with an estimated incidence of new cases of 70–140/million per year [7,8]. Further, 1000 new patients are diagnosed with ESRD each year, and the most common cause of renal failure in Sudanese people (53.6%) is unknown [9,10]. In general, the number of patients with ESRD (who require dialysis or renal transplant to survive) is increasing, and it is becoming a major public health concern worldwide [11].

In 2007, there were over 1.6 million dialysis patients and half a million renal transplant recipients worldwide [12]. Renal transplantation remains the most effective CRD/ESRD treatment, accounting for 28% of total renal therapy in Sudan [12,13]. Transplant rejection can be hyperacute (minutes to hours), acute (days to weeks), or chronic (months to years) [14]. The International Society of Nephrology analyzed data from 182 countries and reported a rejection rate of 59% [15]. Another study conducted in Iran identified the clinical causes of renal allograft nephrectomy, with chronic rejection (38%) being the most common cause [16,17]. Furthermore, a study performed between November 2011 and 2015 at Sharg El-Neel Hospital in Khartoum, Sudan, discovered that the rate of acute rejection was 10.4% [9]. Human leukocyte antigen (HLA) typing is an important step in transplantation, and a well-matched donor is critical for successful transplantation [17].

HLA genes are located on chromosome 6p (short arm) in the distal portion of the 21.3 band, one of the most polymorphic and gene-dense regions [18,19]. HLA complex genes and their protein products are divided into three classes based on their tissue distribution, structure, and function [20]. MHC class II antigens encoded by the HLA-DM, -DO, -DP, -DQ, and -DR loci, and their products are included in the immunoglobulin supergene family [21]. HLA-DR is a heterodimer comprising an alpha chain (DRA) and a beta chain (DRB) [19]. According to the IPD-IMGT/HLA database, HLA-DRB1 is the most polymorphic in class II of this system, with 3298 alleles in September 2022 (https://www.ebi.ac.uk/ipd/imgt/hla/about/statistics/) (accessed on 25 September 2022). The HLA-DRB1 gene is located in GRCh38.p12 (Genome Reference Consortium Human Build 38.p12) coordinates 32,578,775 to 32,589,848, has five introns, and is encoded by six exons [22]. Exon one encodes the leader peptide, exons two and three encode the two extracellular domains, exon four encodes the transmembrane domain, and exon five encodes the cytoplasmic tail (https://www.ncbi.nlm.nih.gov/gene/3123) (accessed on 25 September 2022 [23]. Many studies emphasized the importance of exon 2 as it encodes antigen-binding sites, contains the most pathological single nucleotide variants (SNVs), and is commonly included in high throughput HLA-typing commercial kits [24,25,26,27].

Several benefits are associated with HLA matching in organ transplants, such as kidneys, including improved graft function, reduced the incidence of acute or chronic rejection, extended graft survival, and the potential for reduced immunosuppression [28]. It was reported that patient-donor matching of HLA determinants lowers the risks of chronic and acute GVHD (graft-versus-host disease) [29]. Early studies indicated that HLA-DRB1 mismatch is a particular risk factor for rejection and is critical in the first six months after transplantation [24,27,30,31]. As a result of the realization that HLA plays a significant role in transplantation, the use of HLA typing in transplantation has seen numerous advancements [32,33]. Owing to this development, HLA typing has progressed from identifying HLA proteins to identifying HLA gene variations [31]. The 1000 Genomes Project provides an in-depth analysis of common genetic variations (single nucleotide polymorphisms (SNPs) and Insertions–deletions (indels)) in humans and their association with diseases [34]. HLA variants are strongly linked to various diseases and organ transplantation [22,31,35,36]. SNPs are single nucleotide variants (SNVs) in DNA sequences with a population allele frequency of 1% or higher [37,38]. SNVs can be found in both the coding and non-coding regions of the human genome [39]. Non-synonymous SNVs are a type of single variant that represent amino acid substitutions and protein variations [40]. Previous studies have indicated that nsSNPs account for approximately half of the mutations involved in various genetic diseases [41]. Indels are another type of significant genomic variant that are insertions or deletions of one or more DNA nucleotides [42]. The current study aimed to identify functional or marker genetic variants within HLA-DRB1 exon 2 in patients with renal transplant status (acceptance and rejection).

## 2. Materials and Methods

### 2.1. Study Design and Samples Information

This hospital-based case-control study was conducted at Ahmed Gasim and Ibn Sena Hospitals. Blood samples were collected from March to September 2021 using a convenience sampling method. Samples were collected from individuals of any age and sex who had received a renal transplant, regardless of whether they developed graft rejection (acute, hyperacute, or chronic) or acceptance within the first six months. Participants who had their renal transplants rejected because of medical errors or negligence were excluded. The total sample size was 60, divided equally into three groups. The first group included participants who had graft acceptance for more than the first six months. The second group of participants had graft rejection in the first six months, and the third group was the control group. The study was conducted in accordance with the guidelines of the Declaration of Helsinki and approved by the Institutional Ethics Committee of the National University-Sudan under approval No. NU170220219 (date of approval: 17 February 2021). Written informed consent was obtained from the patient(s) for their anonymized information to be published in this article.

### 2.2. DNA Isolation, Amplification, and Sequencing

Genomic DNA was extracted from the blood samples using QiaAmp blood extraction kits according to the manufacturer’s instructions (Qiagen, Hilden, Germany). The extracted DNA was tested for quality using a NanoDrop spectrophotometer (Implen, München, Germany) and stored at −20 °C until molecular analysis.

The following primers were used to amplify the human HLA-DRB1 gene (target region): Forward primer: 5′GTG CTC TCA GAA CTG CTT GC 3′, and reverse primer: 5′ CCT CAG GAA GAC GGA GGA TGA 3′. The PCR reaction mixture contained 1 µL of the extracted DNA added to 4 µL PCR master mix (Solis Biodyne, Tartu, Estonia) containing 1 U DNA polymerase, 12.5 mM MgCl2, and 4 mM dNTPs. The PCR thermal conditions were as follows: Initial denaturation at 94 °C for 5 min, followed by 35 cycles of denaturation at 94 °C for 30 s, annealing at 57 °C for 30 s, extension at 72 °C for 30 s, and a final extension step at 72 °C for 10 min. The thermal conditions were determined using a 2721 Thermocycler (Applied Biosystems, Thermo Fisher Scientific, Budapest, Hungary). Following PCR, the amplicons were visualized using 2% gel electrophoresis (Major Sciences, London, United Kingdom) by applying the PCR product to an electrical current adjusted to 100 V and 70 A for one h.

The amplified PCR amplicons were sequenced in duplicate based on both directions’ primers by the Sanger dideoxynucleotide chain-termination sequencing method using a 3730XL DNA analyzer (Applied Biosystems, Waltham, MA, USA) by Macrogen (Macrogen Inc., Amsterdam, The Netherlands).

### 2.3. Sequences and Variants Analysis Using Bioinformatics

DNA sequencing results of the 60 samples were obtained as AB1 files. Initially, the Chromatogram Explorer program (version 5.0.2.3) was used to assess the overall quality of the sequences (read Phred quality score), trim low-quality ends, and convert AB1 to FASTA formats [43]. Subsequently, the Basic Local Alignment Search Tool (BLAST) algorithm was used to check the specificity of these sequences by comparing them to the Homo sapiens genome (GRCh38.p12) using the Ensembl genome browser (https://www.ensembl.org/index.html) (accessed on 30 September 2022) [44]. Exon 2 regions were manually extracted from the high-quality sequences for further analysis. All sequences, including the reference (target region), were manually prepared and submitted for Multiple Sequence Alignment (MSA) using CLC genomics workbench program version 21.0.5. CLC is a Qiagen-bioinformatics commercial analysis and visualization product (https://www.qiagenbioinformatics.com/) (accessed on 5 October 2022). The gap cost parameters of the alignment algorithm were as follows: Gap open 10.0, gap extension 1.0, and end gap cost. To generate MSA, the CLC employs a progressive alignment algorithm [45]. Organizing sequence data in MSAs can reveal conserved and variable sites (variants or mutations) [46]. Variants were manually extracted from MSA and prepared for further analysis.

**Figure 1:**
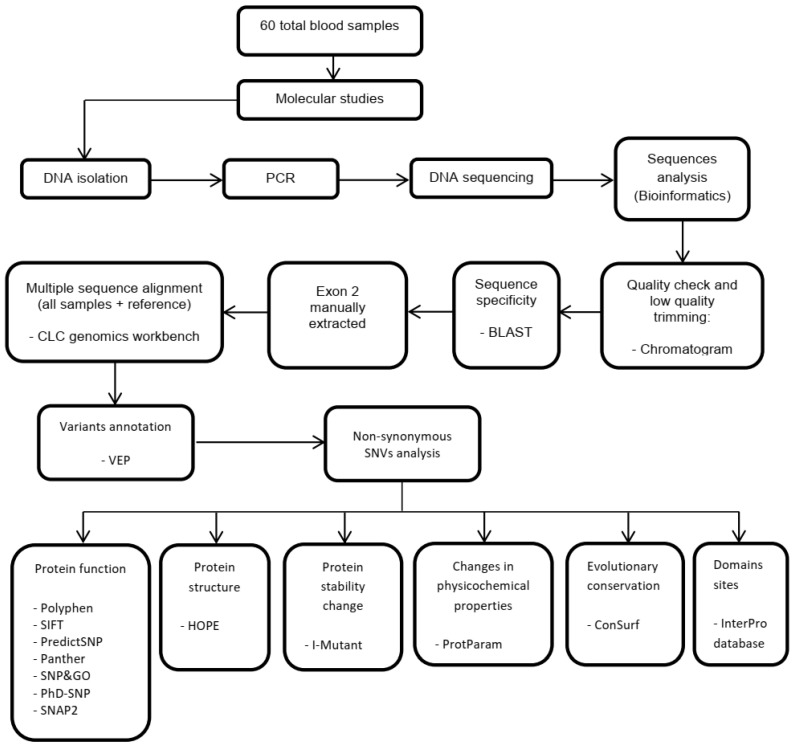
Flowchart of the work methodology. The names that appear after the symbol “-” represent prediction tools.

The chromosomal location of the detected variants was initially submitted to the Ensembl Variant Effect Predictor (VEP) [47]. VEP can annotate, analyze, and prioritize genomic variants in both coding and non-coding regions. VEP was used to determine the variants’ availability, frequency, and amino acid positions. Non-synonymous SNVs were then submitted sequentially to SIFT [48], PolyPhen-2 [49], PredictSNP [50], PANTHER [51], SNP&GO [52], SNAP2 [53], and PhD-SNP [54] tools to differentiate between functional (deleterious) and non-functional nsSNVs. The I-mutant server was used to determine whether nsSNVs affected protein stability [55]. The HOPE server was used to evaluate the effects of nsSNVs on protein structure [56]. The location of the domain and high evolutionary conservation were then determined using the InterPro and Consurf servers [57,58]. Moreover, the ProtParam server was used to assess the impact of nsSNVs on protein physicochemical parameters [59]. Finally, the STRING database (Version 11.5) was used to predict associations between HLA-DRB1 and most related proteins to construct a protein-protein network based on physical interactions and functional associations [60]. The study methodology is summarized in Figure 1. 

The following are websites for the previous tools: VEP https://www.ensembl.org/info/docs/tools/vep/index.html, (accessed on 7 October 2022), SIFT https://sift.bii.a-star.edu.sg/, (accessed on 7 October 2022). PolyPhen-2 http://genetics.bwh.harvard.edu/pph2/, (accessed on 9 October 2022), PredictSNP tool https://loschmidt.chemi.muni.cz/predictsnp/, (accessed on 12 October 2022), PANTHER http://www.pantherdb.org/tools/csnpScoreForm.jsp, (accessed on 15 October 2022), SNP&GO https://snps.biofold.org/snps-and-go/snps-and-go.html, (accessed on 20 October 2022), SNAP2 https://www.rostlab.org/services/snap/, (accessed on 23 October 2022), PhD-SNP https://snps.biofold.org/phd-snp/phd-snp.html, (accessed on 27 October 2022), I-mutant v3.0 http://gpcr2.biocomp.unibo.it/cgi/predictors/I-Mutant3.0/I-Mutant3.0.cgi, (accessed on 1 November 2022), HOPE https://www3.cmbi.umcn.nl/hope/, (accessed on 5 November 2022), InterPro database https://www.ebi.ac.uk/interpro/, (accessed on 10 November 2022), Consurf https://consurf.tau.ac.il/, (accessed on 13 November 2022) ProtParam https://web.expasy.org/protparam/, (accessed on 17 November 2022), STRING database https://string-db.org/ (accessed on 20 November 2022).

## 3. Results

This study used DNA from 40 samples (acceptance and rejection) and 20 controls to target the HLA-DRB1 exon 2. BLAST revealed that all DNA quality-checked sequences showed high similarity (>99%) and specificity for the HLA-DRB1 target region. Multiple sequence alignments revealed seven SNVs in ten samples and controls. The Ensembl variant effect predictor was used to collect broad information on seven SNVs, two of which were novel (Table 1). 

Three of the seven detected SNVs were non-synonymous and were only located in the rejected samples (R3, R9, and R16), whereas the remaining were synonymous (Table 1). To identify the deleterious effects of nsSNVs at the functional level, seven different tools (SIFT, PolyPhen, PredictSNP, PANTHER, SNP&GO, SNAP2, and PhD-SNP) with different prediction algorithms were used. Two nsSNVs (K41N and R109S) were predicted to be pathogenic by all seven tools, whereas the third (Y59H) was predicted by only four (Table 2). Furthermore, the I-mutant server predicted that all nsSNVs would affect the protein stability (Table 2). 

**Figure 2:**
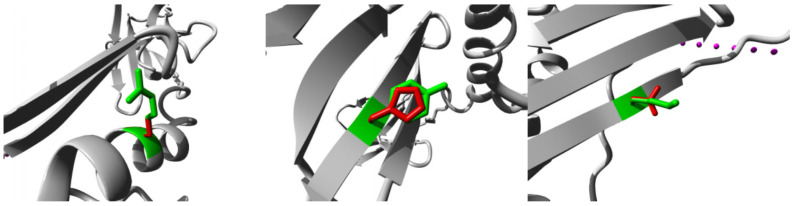
Structural alteration by HOPE server. The protein is shown in grey, the wild-type residue in green, and the variant residue in red. The structures from left to right represent R109S, Y59H, and K41N variants.

At the structural level (using the HOPE server), the new residues differed in size, charge, and hydrophobicity. The new residues also influenced hydrogen bond formation, ionic interactions, multimer contacts or interactions, and the function of their region (Table 3 and Figure 2). 

**Figure 3:**
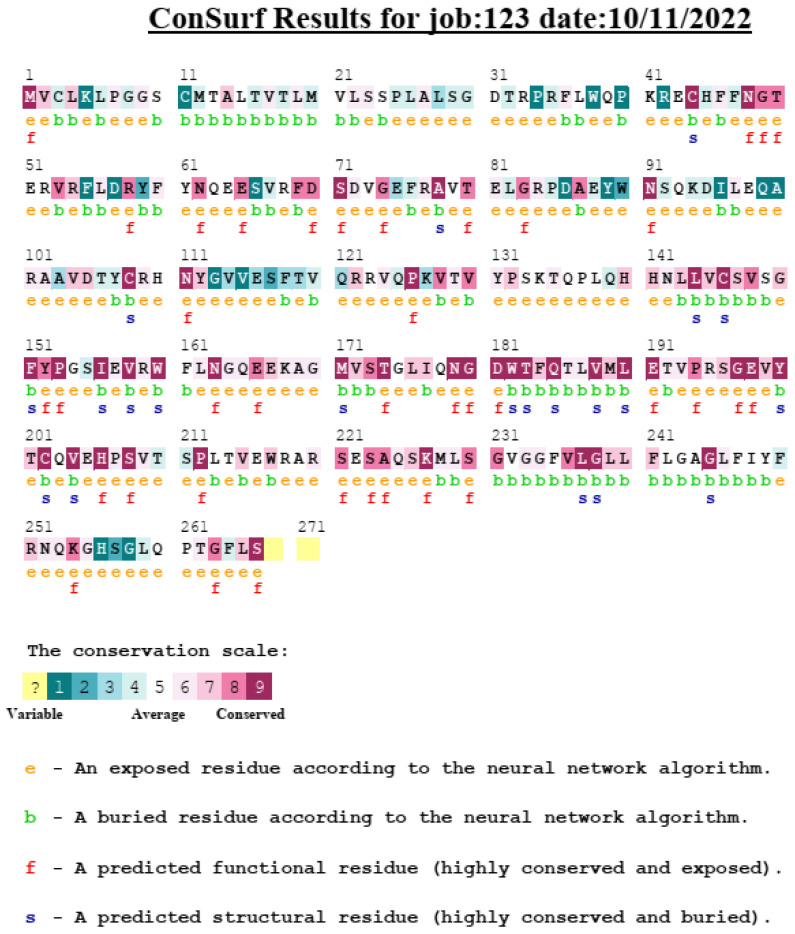
Evolutionary conservancy of *HLA-DRB1* produced by Consurf server.

The Consurf server and InterPro database were used to predict the locations of variants in evolutionarily conserved and domain regions. The three nsSNVs K41N, Y59H, and R109S received scores of 5, 1, and 6, respectively, indicating that they were average, variable, and conserved, respectively (Figure 3). 

Additionally, two of the three nsSNVs (Y59H and R109S) were discovered in the MHC II b N domain (MHC class II, beta chain, N-terminal) with the accession number IPR000353. All nsSNVs demonstrated changes in the overall protein physicochemical parameters. The properties altered by all three nsSNVs were the molecular weight, theoretical isoelectric point (pI), atomic composition, instability index, and GRAVY (Table 4). 

**Figure 4:**
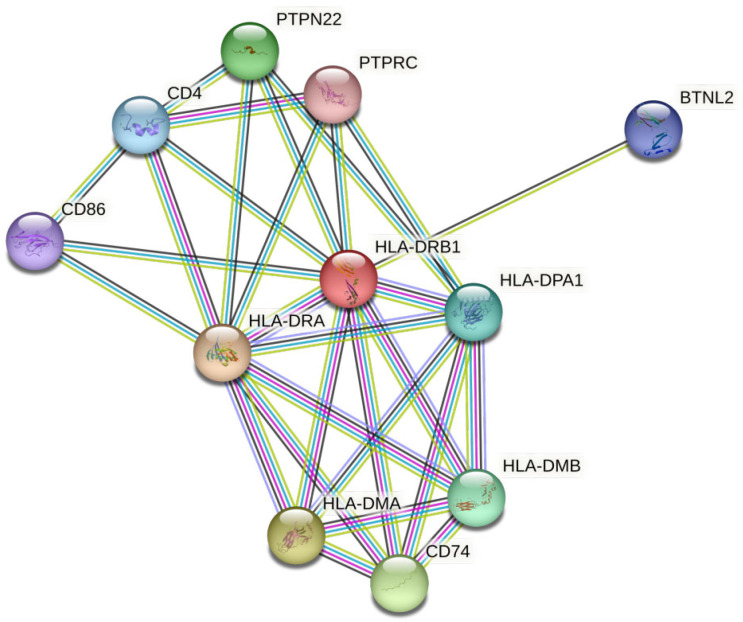
Protein-protein interaction network of HLA-DRB1 predicted by the STRING database. The current illustration depicts the ten proteins with the highest total probability score. The red, green, blue, purple, yellow, light blue, and black lines indicate the presence of fusion, neighborhood, co-occurrence, experimental, text mining, database, and co-expression evidence, respectively.

HLA-DRB1 interacts with HLA-DRA, HLA-DMA, CD74, HLA-DMB, HLA-DPA1, CD4, BTNL2, and CD86, in that order, according to the protein-protein interaction network (Figure 4). Finally, no significant variants that had an impact on or worked as markers for transplant acceptance samples were found (Table 1).

## 4. Discussion

Chronic renal diseases in general and end-stage renal disease in particular are major health concerns in Sudan and around the world [1,6,7,8,9]. The growing number of ESRD patients places strain on both individuals (costs of dialysis or transplantation) and governments (increasing the financial burden of health care) [61]. Renal transplantation remains the most effective ESRD treatment, and HLA typing is the most important test in this process [13,17]. The HLA region is extremely diverse, and HLA-DRB1 is the most polymorphic in class II of this system [19,62]. HLA-DRB1 protein is significantly associated with graft survival, particularly in the first six months after transplantation [27,28]. HLA-DRB1 exon 2 is important because it encodes antigen-binding sites and contains the most pathological single nucleotide variants (SNVs) [24,25,26,27]. The present study aimed to identify functional or marker genetic variants in HLA-DRB1 exon 2 in renal transplant recipients (acceptance and rejection) using sequencing technology. 

This study included 60 DNA samples (from various families) divided into three equal groups: Control, renal transplant rejection, and acceptance. The alignment algorithm identified seven SNVs at six locations, three of which were non-synonymous and could have functional consequences. Two of the three nsSNVs were found in the public domain archive of simple genetic polymorphisms (https://www.ncbi.nlm.nih.gov/snp/), (accessed on 20 December 2022), whereas the third was not found. Several algorithms and methods have been used to assess the impact of these three nsSNVs on protein function and stability indices. They discovered that two were high-impact, indicating deleterious effects by all tools, and the third had a lower impact. Previous (the preceding three decades) and recent studies have shown a close relationship between HLA mismatch and transplantation graft rejection [63,64]. Previous findings have indicated that R109S and K41N variants may have a greater impact on protein function than the Y59H variant. None of the three detected variants corresponded to an in silico study conducted in 2014 by Hassan et al., who identified the most deleterious nsSNVs in the human HLA-DRB1 gene [25]. The nsSNVs discovered in this study were submitted to dbSNP after Hassan et al.’s study, resulting in incompatibility. https://www.ncbi.nlm.nih.gov/snp/rs750986830#submissions, https://www.ncbi.nlm.nih.gov/snp/rs11554462#submissions (accessed on 5 April 2023).

At the structural level, all the variant amino acids showed differences in physical properties, bond formation, and a variety of interactions. The HOPE server predicted that the three nsSNVs are situated in a unique region called Beta-1. According to the Universal Protein Resource, the beta-1 region is a structural part of the peptide-binding cleft of HLA-DRB1, consisting of 94 amino acids. Additionally, beta-1 interacts with the T-cell receptors CDR2 and CDR3 (complementarity-determining regions 2 and 3) alpha domains through hydrogen bonds. https://www.uniprot.org/uniprotkb/P01911/entry (accessed on 15 December 2022). Differences in amino acid properties can disrupt this region (beta-1) and its function. According to evolutionary conservation, the K41N, Y59H, and R109S variants were average, variable, and conserved, respectively. Two of these (Y59H and R109S) were found in the MHC II b N domain. Compared to other variants, the R109S variant may have the greatest impact. This is because of its conserved nature, main domain location, and pathogenic impact on protein structure, function, and stability.

In terms of physicochemical properties, the HOPE tool, as previously mentioned, revealed differences in the residue levels, whereas ProtParam indicated that the variants caused changes in the entire protein. All the nsSNVs detected agreed to alter the molecular weight, theoretical pi (isoelectric point), atomic composition, and GRAVY of the protein. The extinction coefficients and total positive charge were altered in a few variants, but the aliphatic index and total negative charge remained the same. In general, all the nsSNVs had nearly equal effects on the overall physicochemical properties of the protein. Most proteins function consecutively with other proteins in living organisms, and protein-protein interaction studies provide crucial information for understanding the complicated biological processes that occur in live cells [65,66]. Thus, to gain a better understanding, a network of protein-protein interactions (PPIs) was constructed using the STRING database. Deleterious variants in the HLA-DRB1 protein could disrupt its interaction with confidence interaction proteins. Only one synonymous SNV (K41K) was found in the transplant acceptance samples in the current study. The K41K variant could not be identified as an acceptance marker because it appeared in both acceptance and rejection cases.

## Figures and Tables

**Table 1:** General information of detected SNVs.

Serial No.	SMPs Code No.	N.P Exon 2	Chr. Location	Variants	Variants Type	SNV AVAIL.	AA Change	Variants Allele Frequencies ALL/African			ClinVar
								1000 Genome	GnomAD Genomes	NCBI ALFA	
1.	R3/C12	5	6:32584113	C/A	SNV	Novel	R122R	-	-	-	-
2.	R20	14	6:32,584,122	T/C	SNV	rs1,136,782	T119T	0.065/0.129	0.001/0.002	0.006/0.013	-
3.	R16	44	6:32,584,152	T/A	SNV	rs750,986,830	R109S	-	-	-	-
4.	R9	196	6:32,584,304	A/G	SNV	rs11,554,462	Y59H	0.072/0.167	0.169/0.318	0.166/0.290	-
5.	R3/C10	242	6:32,584,350	C/T	SNV	rs17,885,011	E43E	0.063/0.060	0.112/0.069	0.096/0.071	-
6.	A7/R12	248	6:32,584,356	C/T	SNV	rs17,887,028	K41K	-	-	0.000/0.000	-
7.	R3	248	6:32,584,356	C/A	SNV	Novel	K41N	-	-	-	-

SMPs code No.: The code number of samples containing genetic variant(s), which are as follows: Rejection (R), acceptance (A), and control (C). N.P Exon 2: Position of a nucleotide in Exon 2. Chr. Location: Chromosomes Location. SNV AVAIL.: SNV availability in the Single Nucleotide Polymorphism database (dbSNP). AA Change: Type and location of amino acid changed. ClinVar: ClinVar database clinical significance records (https://www.ncbi.nlm.nih.gov/clinvar/) (accessed on 1 December 2022). The symbol “-” refers to unavailable data.

**Table 2:** The functional effect and stability index of non-synonymous detected SNVs.

Variant Description	SNV ID	AA Change	SIFT	PolyPhen-2	PredictSNP	PANTHER	SNP&GO	SNAP2	PhD-SNP	I-Mutant
chr6(GRCh38.p12):32584152T>A	rs750986830	R109S	Deleterious (0.0)	Probably damaging (0.999)	Deleterious	Possibly damaging	Disease (0.908)	Effect (47)	Disease	Decrease (−1.29)
chr6(GRCh38.p12):32584304A>G	rs11554462	Y59H	Tolerated (0.3)	Possibly damaging (0.833)	Neutral	Probably damaging	Disease (0.551)	Effect (52)	Neutral	Decrease (−1.60)
chr6(GRCh38.p12):32584356C>A	Novel	K41N	Deleterious (0.01)	Probably damaging (0.993)	Deleterious	Possibly damaging	Disease (0.785)	Effect (44)	Disease	Decrease (−0.34)

Variant Description: In variant call format (VCF). SNV ID: Accession number in dbSNP. AA Change: Type and location of amino acid changed. The value of the predicted score is represented by the numbers between the brackets.

**Table 3:** Structural effects of nsSNVs in a protein sequence using the HOPE server.

Distinctions and Characteristics	rs750986830 R109S		rs11554462 Y59H		Novel K41N	
	Arginine	Serine	Tyrosine	Histidine	Lysine	Asparagine
Schematic structures	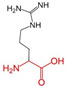	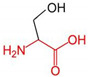	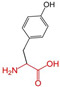	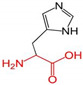	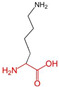	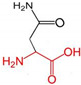
Size	large	Small	large	Small	large	Small
Charge	Positive	Neutral	-	-	Positive	Neutral
Hydrophobicity-value	Less hydrophobic	More hydrophobic	More hydrophobic	Less hydrophobic	-	-
Contacts	The wild-type residue forms eight hydrogen bonds and one salt bridge with other residues.	The mutant-type has an impact on the original’s hydrogen bond formation, binding site, and ionic interactions.	The wild-type residue forms a hydrogen bond with eight residues.	The mutant-type has an impact on the original’s hydrogen bond formation.	The wild-type forms a hydrogen bond, a salt bridge with one residue, and is involved in multimer contacts.	The mutant type affects hydrogen bond formation, ionic interaction, and the development of multimer interactions.
Structure	The mutation is located within a stretch of residues annotated in UniProt as a special region: Beta-1. The differences in amino acid properties can disturb this region and disturb its function.					

UniProt: Universal database of protein (https://www.uniprot.org/) (accessed on 10 December 2022). The symbol “-” refers to unavailable data.

**Table 4:** The effect of nsSNVs on HLA-DRB1’ protein physicochemical parameters.

Reference & Variants	Molecular Weight	Theoretical pI	Atomic Composition	Total −ve	Total +ve	Extinction Coefficients	Instability Index	Aliphatic Index	GRAVY
Reference	29966.14	7.64	C_1342_H_2068_N_368_O_389_S_12_	25	26	41285	48.92	77.93	−0.207
R109S	29897.03	7.00	C_1339_H_2061_N_365_O_390_S_12_	25	25	41285	48.20	77.93	−0.193
Y59H	29940.10	7.66	C_1339_H_2066_N_370_O_388_S_12_	25	26	39795	49.54	77.93	−0.214
K41N	29952.07	7.00	C_1340_H_2062_N_368_O_390_S_12_	25	25	41285	47.70	77.93	−0.205

The accession number for the reference sequence is P01911 (https://www.uniprot.org/) (accessed on 15 December 2022). Total –ve: Total negatively charged residues. Total +ve: Total positively charged residues. GRAVY: Grand average of hydropathicity index. The parameters that have been changed compared to the reference are highlighted in bold.

## Data Availability

The sequences data that support the findings of this study are available in [National Center for Biotechnology Information-genebank database] at [https://www.ncbi.nlm.nih.gov/nucleotide/, accessed on 10 April 2023], accession numbers [OQ747803-OQ747862].
